# Cadmium Level, Glycemic Control, and Indices of Renal Function in Treated Type II Diabetics: Implications for Polluted Environments

**DOI:** 10.3389/fpubh.2016.00114

**Published:** 2016-06-13

**Authors:** John I. Anetor, Chukwuemelie Z. Uche, Emmanuel B. Ayita, Solomon K. Adedapo, Jokotade O. Adeleye, Gloria O. Anetor, Sola K. Akinlade

**Affiliations:** ^1^Micronutrient Metabolism and Toxicology Unit, Department of Chemical, College of Medicine, University of Ibadan, Ibadan, Nigeria; ^2^Endocrinology and Metabolic Research Unit, Department of Chemical Pathology, College of Medicine, University of Ibadan, Ibadan, Nigeria; ^3^Endocrinology Unit, Department of Medicine, College of Medicine, University of Ibadan, Ibadan, Nigeria; ^4^Public Health Unit, School of Health Sciences, National Open University of Nigeria, Lagos, Nigeria

**Keywords:** cadmium–zinc interaction, glycated hemoglobin, diabetes mellitus, renal function and environmental health

## Abstract

Cadmium (Cd) has recently emerged as a major concern not only in environmental toxicology but also in metabolic diseases such as diabetes mellitus and its complications. Conflicting data aside, these studies have not been examined in a clinical population undergoing management as well as possible modulation by the prominent metabolic antagonist of Cd such as zinc (Zn). This study examined the relationship between cadmium levels, glycemic control, and renal pathology in established type II diabetic patients with focus on populations exposed to modern environmental health hazards (MEHHs). Sixty-five participants, consisting of 45 type-2 diabetics and 20 non-diabetics were enrolled for the study, mean age 61.51 ± 5.27 years. Glycated hemoglobin (HbA_1c_) was used to classify them into three sub-groups: (A) good glycemic control (44.4%), (B) fair glycemic control (24.4%), and (C) poor glycemic control (31.1%). Plasma levels of glucose, Cd, Zn, HbA_1c_, creatinine, urinary creatinine, microalbuminuria, and estimated glomerular filtration rate (eGFR) were determined in all participants using standard methods. Fasting plasma glucose was higher in diabetics than in non-diabetics (*p* = 0.000) as well as Zn level, though not significantly. Interestingly, Cd level, Cd/Zn ratio, and urinary creatinine were significantly lower in diabetics than in non-diabetics. The group with poor glycemic control (C) had significantly higher Cd level compared to the one with good glycemic control (group A). The renal function revealed that microalbuminuria and urinary albumin/creatinine ratio (UACR) was significantly higher in diabetics than in non-diabetics, while eGFR was found to be similar in both diabetics and non-diabetics. UACR inversely correlated with Cd level, while plasma creatinine level positively correlated with Cd but not significantly. Correlation between Cd and HbA_1c_ revealed non-significant inverse correlation (*r* = −0.007; *p* > 0.05), while Zn showed a significant inverse correlation with Cd (*r* = −0.317; *p* < 0.014). The lower Cd level in diabetics compared to non-diabetics probably reflects the modulating effect of Zn in treated diabetics given nutritional education in addition to their regular regime, including good sources of Zn. The renal insufficiency with increasing Cd level may suggest that the progression of renal impairment may not be responsive to the putative modulating effect of Zn.

## Introduction

Cadmium (Cd) is a non-essential (toxic) metal that is ubiquitous in the environment. It is a persistent non-biodegradable toxicant with a long biological half life of about 10–30 years (1). It is a known metabolic antagonist of zinc, a very essential micronutrient that is vital in many intermediary metabolic and molecular events (2, 3). Human exposure to Cd has been associated with a number of biological and medical effects that makes it of critical importance in biology and medicine. Cadmium has been traditionally implicated in renal and hepatic diseases and probably more internationally as a factor in itai-itai disease, which is characterized by renal tubular dysfunction, osteomalacia, and pseudo fractures. Very recently, an association between cadmium and metabolic diseases has been reported (4).

Indeed, the rising global incidence of diabetes mellitus (DM) has been correlated with the rising level of environmental pollution, suggesting that an increase in diabetes may likely occur even if the prevalence of obesity is held constant (5). A growing body of knowledge consistently suggests the involvement of environmental factors in the etiology of type II diabetes, and this is also being gradually accepted as at least in part an explanation for the global increasing rate of type II DM in both the developed and the developing countries (6). Although factors such as obesity, body mass index (BMI), diet, and lifestyle have been primarily identified as risk factors for diabetes, environmental pollutants, of which cadmium is a prime candidate, have been consistently linked (7).

Epidemiological studies have shown that there is an association between Cd exposure, incidence, severity, and complications of DM (8). A number of mechanisms have been adduced for this association; Cd apart from being pancreatotoxic also accumulates in the pancreas, where it serves as a metabolic antagonist of Zn, which potentiates insulin action among others (9).

Experimental studies have shown that Cd elevates fasting blood glucose before overt signs of renal dysfunction are evident and that Cd acts synergistically with chronic hyperglycemia seen in diabetes nephropathy (8). Cadmium is thus considered to play a crucial role in the progression of DM-related kidney disease and more importantly, due to its accumulation in other organs, such as the eye, liver, bone, pancreas, and nerves (10–12). Any substance that has been implicated in such a diverse group of pathological conditions particularly in an important one like DM should attract attention and stimulate research.

In rapidly industrializing developing countries, environmental Cd level and the incidence of type II diabetes are on the increase as well as the incidence of type II DM (13). This may be due to the extensive use of cadmium in industries and its widespread dispersion in the environment, particularly in Nigeria, where owing to progressive industrialization and often poor regulation of chemical exposure, may be higher and perhaps of greater severity. Hence, a possible link between cadmium exposure, DM, and diabetic nephropathy deserve exploration. Various studies have focused on the early stage of cadmium-induced kidney injury in exposed human populations without considering DM (14, 15). Few studies have examined clinical cases along with one of the most important long-term complications of DM and diabetes nephropathy. Thus, this study is designed to examine the relationship between cadmium levels, glycemic control, and renal pathology in established T2DM patients in Ibadan, South Western Nigeria.

## Materials and Methods

### Subjects

A total of 65 participants were recruited into this case–control study. They are comprised of 45 patients with T2DM, recruited from the Metabolic Research Ward (MRW) of the University College Hospital (UCH), Ibadan, while 20 apparently healthy (non-diabetic) individuals age-matched with the cases served as controls. All T2DM were on diet and/or oral hypoglycemic drugs. They also received regular health education talks. The percentage level of glycated hemoglobin was used to classify the T2MD patients into three sub-groups: good control (A), fair control (B), and poor control (C). Smokers, alcoholics, pregnant women, lactating mothers, and those who did not give informed consent were excluded from the study. Information on demography was obtained using a semi-structured questionnaire. The study was approved by the UI/UCH Joint Ethics Committee.

### Anthropometric Measurement

#### Height

This was measured using a stadiometer, and the readings recorded in meters.

#### Body Weight

This was determined with Omron weighing scale (HBF, 202) placed on a flat surface, and the readings were recorded to the nearest 0.5 kg.

#### Body Mass Index

This was calculated as the ratio of body weight to the square of the height in meter; BMI (kilograms per square meter) = body weight (kilograms)/height^2^ (square meter).

#### Blood Pressure

The blood pressure (BP) of the participants was measured by using standard mercury sphygmomanometer after 10 min of rest. The readings were recorded to the nearest (millimeter of mercury). This was taken for each patient at sitting position.

### Sample Collection

#### Blood Samples

After an overnight fast of at least 10 h, 10 ml of venous blood was obtained from the ante cubical fossa of each participant using a sterile needle and syringe and dispensed into appropriate sample bottles. Two milliliters were dispensed into EDTA bottle for HbA_1c_ determination. Five milliliters were dispensed into lithium heparin bottle for cadmium, creatinine, and zinc determinations, while the rest was dispensed into fluoride oxalate bottle for glucose estimation. Plasma samples were appropriately obtained and stored at −20°C until analysis. Whole blood used for HbA_1c_ analysis was stored at 4°C for 10 days.

#### Urine Samples

About 10 ml of spot urine sample was obtained from each participant into sterile universal bottles and stored at −4°C until analyzed. Urine samples were used for the determination of urinary microalbumin and creatinine.

### Biochemical Assays

#### Determination of Plasma Cadmium and Zinc

Atomic absorption spectrophotometric (AAS) technique was used for the determination of cadmium and zinc using Bulk Scientific, 210 VGP model Atomic Absorption Spectrophotometer (Germany). Plasma Cd was determined following the method of Robert and Clark (16); plasma was used instead of serum to ensure adequate stoichiometric relationship to Zn. Zinc was determined according to the method of Smith et al. (17).

#### Plasma Glucose Determination

Fasting plasma glucose (FPG) was determined using glucose oxidase method (18), using Dialab Diagnostic reagent kits (Gessellschaft m.b.H A-1160 Wienpanikengasse, Austria).

#### Glycosylated Hemoglobin (HbA_1c_) Determination

HbA_1c_ was determined using boronate affinity assay method as described by Jeppsson et al. (19). NycoCard HbA_1c_ analyzer, which is a rapid *in vitro* method for the measurement of glycated hemoglobin (% HbA_1c_) in human whole blood, was used.

#### Determination of Microalbumin

Urinary microalbumin was determined using immunoturbidimetric assay method (20).

#### Determination of Plasma Creatinine

Urinary and plasma creatinine were determined by colorimetric method (Jaffe’s reaction without deproteinization) according to the methods of Bartels et al. (21) and Henry et al. (22).

#### Urinary Albumin/Creatinine Ratio

This was calculated using the formula below:

Microalbumin (mg/dl)/creatinine (mg/dl)×1000=UACR (mg/g).

#### Estimated Glomerular Filtration Rate

This was calculated using the Modification of Diet in Renal Disease (MDRD) study formula as given below (23):

$$\begin{array}{c} \text{MDRD}=186\;\times \;{\left(\text{plasma creatinine}\right)}^{-1.154}\times {\left(\text{age}\right)}^{-0.203}\times \;(1.212\text{ if black})\;\times \;(0.742\text{ if female)} \end{array}$$

### Statistical Analyses

Data analysis was conducted out using SPSS version 21.0. Results were expressed as mean ± SEM. Student’s *t*-test was used to determine significant differences between the means values; analysis of variance (ANOVA) was used to compare means across groups. Relationship between parameters was assessed using Pearson’s correlation coefficient. *p* value of <0.05 was considered significant.

## Results

Out of the total 45 diabetics studied, 44.4% had good glycemic control, while 24.4% exhibited fair glycemic control and 31.1% demonstrated poor glycemic controlled (Table 1). There were no statistically significant differences found in the mean age, BMI, and systolic and diastolic BP between diabetics and the non-diabetic groups (Table 2).

**Table 1:** **Classification of glycemic control in diabetics**.

Group	HbA_1c_ (%)	Number of subject
Good control	<6.5%	20 (44.4%)
Fairly good control	6.5–7.9%	11 (24.4%)
Poor control	≥8.0%	14 (31.1%)
Total	–	65 (100%)

*HbA_1c_, glycated hemoglobin*.

**Table 2:** **Demographic indices and anthropometric measurements of diabetics and controls**.

Indices	Diabetic (*n* = 45)	Control (*n* = 20)	*t*-value	*p*-value
Age (year)	61.51 ± 8.527	57.35 ± 9.659	1.660	0.106
BMI (kg/m^2^)	26.027 ± 3.864	25.073 ± 3.884	0.915	0.366
SBP (mmHg)	130.78 ± 16.176	135.89 ± 16.295	1.067	0.293
DBP (mmHg)	79.41 ± 9.370	79.17 ± 11.147	0.077	0.939

**n*, number of subjects; BMI, body mass index; SPB, systolic blood pressure; DBP, diastolic blood pressure*.

Urinary creatinine was significantly lower in diabetics than in non-diabetics (*p* < 0.017). The microalbumin and albumin/creatinine ratio, in contrast, was significantly higher in diabetics than in non-diabetics. All other parameters were similar (*p* > 0.05) (Table 3). Cadmium and cadmium–zinc ratio demonstrated significantly lower levels in diabetics than non-diabetics and were significantly lower in diabetics than in non-diabetics (*p* < 0.001). Contrary to expectation, Zn level was not significantly higher in diabetics than in non-diabetics, while FPG level was significantly higher in diabetics than in non-diabetics (*p* < 0.001) (Table 4).

**Table 3:** **Plasma creatinine and urinary creatinine, microalbumin, albumin–creatinine ratio (ACR), and estimated glomerular filtration rate (eGFR) in diabetics and controls**.

Indices	Diabetics (*n* = 45)	Controls (*n* = 20)	*t*-value	*p*-value
Plasma creatinine (mg/dl)	1.34 ± 0.29	1.43 ± 0.18	1.47	0.146
Urinary creatinine (mg/dl)	85.33 ± 32.91	115.25 ± 47.58	2.554	0.017*
Microalbumin (mg/dl)	1.49 ± 0.60	1.03 ± 0.53	3.109	0.003*
Albumin–creatinine ratio (mg/g)	17.44 ± 18.08	8.89 ± 11.16	2.709	0.009*
eGFR (ml/min/1.73 m^2^)	59.78 ± 13.83	55.69 ± 9.91	1.354	0.182

*Significant (*p* < 0.05).

**n*, number of participants; eGFR, estimated glomerular filtration rate*.

**Table 4:** **Fasting plasma glucose, cadmium, zinc, and Cd/Zn ratio in diabetics and non-diabetic (controls)**.

	Diabetics (*n* = 45)	Controls (*n* = 20)	*t*-value	*p*-value
FPG (mg/dl)	150.903 ± 79.394	96.620 ± 11.957	4.474	0.000*
Cadmium (μg/dl)	0.050 ± 0.0201	0.095 ± 0.0311	5.763	0.000*
Zinc (μg/dl)	102.51 ± 10.11	98.1 ± 10.76	1.496	0.144
Cd/Zn ratio	0.002 ± 0.000	0.004 ± 0.001	5.485	0.000*

*Significant (*p* < 0.05).

**p*, probability; FPG, fasting plasma glucose*.

Correlation studies showed that cadmium was significantly and inversely correlated with zinc (*r* = −0.317, *p* < 0.01) (Table 5). Cadmium was also inversely correlated with FPG, HbA_1c_, albumin–creatinine ratio, and eGFR, but all did not reach significant levels (*p* > 0.05) in the cases (Table 5). Other parameters were similar.

**Table 5:** **Correlation of cadmium with biochemical indices**.

Parameters	*r*-value	*p*-value
Fasting plasma glucose (mg/dl)	−0.198	0.133
HbA_1c_ (%)	−0.007	0.967
Zinc (μg/dl)	−0.317	0.014*
Plasma creatinine (mg/dl)	0.149	0.258
Urinary albumin–creatinine ratio (mg/g)	−0.172	0.198
eGFR (ml/min/1.73 m^2^)	−0.127	0.338

*Significant (*p* < 0.05).

*HbA_1c_, glycated hemoglobin; eGFR, estimated glomerular filtration rate*.

## Discussion

The contribution to the evolution and complications of metabolic disorders by modern environmental health hazards (MEHHs) is at the moment poorly recognized. But it is a reality we cannot escape from. The contribution of key environment toxicant cadmium to metabolic disorders, such as DM and metabolic bone disease, though referred to in pockets, deserves greater attention to avoid their emergence into problems requiring extreme public health intervention. Type II DM has been associated with a combination of genetic, lifestyle, and environmental factors. Though, factors such as obesity, BMI, diet, and lifestyle have been primarily identified as major risk factors for diabetes, environmental pollutants have also been suggested as possible link to diabetes, of which cadmium has been one of the most strongly implicated (8).

The significant increase in FPG level in diabetics compared to the non-diabetics is not unexpected. It is not surprising as DM is characterized by persistent hyperglycemia and is the most prominent disease related to failure of blood glucose homeostasis. An earlier study from this environment (7) also showed significantly higher fasting blood glucose in diabetics than in non-diabetics.

Interestingly, cadmium was found in this study to be significantly lower in diabetics compared with non-diabetics (*p* = 0.000). This may largely have arisen from the effect of the regular health education given to the diabetics embracing better nutrition including sources of Zn. This contradicts the observation of earlier investigators (7, 24). They reported higher cadmium levels in diabetics compared with the non-diabetics. Our position may be partly attributed to the observed higher zinc level in diabetics compared with non-diabetics though it did not reach significant level. Mutual antagonism is a well-established relationship between cadmium and zinc (25). The two metals are known to compete for common metabolic pathways, such as binding sites on metallothionein, a common binding protein for cadmium and zinc. This implies that as the zinc level is increasing, the cadmium level will be decreasing as evidenced in this study. The metallokinetics may, indeed at least in part, account for the non-significant difference in the diabetics and non-diabetics.

The significant inverse correlation between zinc and cadmium in this study could be reflective of metabolic modulation or due to decreased Zn bioavailability and absorption as a result of the competition of cadmium with zinc. This was also corroborated by the cadmium:zinc ratio, which was significantly lower in diabetics than in non-diabetics. This suggests that zinc may have a profound modulating effect on cadmium toxicity. It has been consistently demonstrated that non-essential toxicant metals mimic essential metals and consequently gain access to, and disrupt, key cellular functions (26, 27). This study appears to have elegantly upheld this seminal observation. It is an important concept that could be explored to ameliorate MEHHs as illustrated between Cd and Zn in this report. The observed significant reduction in urinary creatinine, while microalbumin and albumin:creatinine ratio increased in diabetics compared to non-diabetics (*p* < 0.05), may all be indicative of impaired renal function. Cadmium is an established nephrotoxicant. The combined effect of the lung-term renal impairment of DM and that of Cd may be additive and may be a plausible explanation here. This may lead to accelerated renal impairment in DM in this population. This agrees with the earlier findings of Schrijvers et al. (28) as well as Edwards and Prozialeck (8) who reported that diabetic nephropathy was associated with microalbuminuria. DM is one of the primary risk factors for developing renal impairment globally (29, 30). Both type 1 and type 2 DM may lead to chronic complication of diabetic nephropathy (31). Urine microalbumin and urine albumin creatinine ratio have all been reported as sensitive and early indicators of renal impairment (32). The significantly higher albumin:creatinine ratio was significant in diabetics compared to non-diabetics (*p* = 0.009), and the negative correlation with cadmium, though not significantly (*p* > 0.05), may be partially corroborative of the microalbuminuria data. Although previous studies have associated high blood cadmium level in microalbuminuria, in this study, lower levels of cadmium were found in the diabetics. Studies have shown early adverse health effects at much lower levels of cadmium exposure than previously reported (33).

The significantly raised microalbuminuria seen in the diabetics here may be multifactorial, the cadmium effects with other confounders, such as age and sex, as kidney function declines with age (34), and cadmium, a cumulative toxicant is also known to accumulate with age. The significant positive correlation of BMI with systolic BP (*r* = 0.281; *p* = 0.048) and non-significantly with diastolic BP (*r* = 0.236; *p* = 0.10) probably confirms this well-known relationship in the face of an environmental assault. This is in agreement with the observation of Mungreiphy et al. (35), who reported a significant positive correlation between mean systolic and diastolic BPs among different BMI categories. They suggested that both systolic and diastolic BP increased with increase in BMI level. A number of other investigators have concluded that among many relevant factors, BMI is one of the most important predictors of BP (36–39). This may be partly attributed to the role of body weight in the pathogenesis of hypertension. Recent studies have shown that increase in body weight affects the activities of the adipocytes and the secretion of adipokines (leptin and adiponectin). These adipokines affect the development of hypertension (40). The role of an environmental toxicant, such as Cd which though in the past related to hypertension through sodium exchange mechanisms, is incompletely elucidated.

The similar plasma creatinine, eGFR levels, and indices of renal function found in diabetics and non-diabetics may be due to the ameliorating effect of Zn on Cd. The small sample size of the current study may also be contributory to the equivocal findings in certain respect. A larger sample size may bring out the true situation of the role of this environmental pollutant on metabolic disease and in organs that have metabolic roles and lead to metabolic disorders when diseased, such as the kidney. This may lead to renal osteodystrophy, a renal induced bone disease that may be secondary to the adverse effect of Cd on the kidney.

Good glycemic control is required for the proper management of DM. Studies have shown an association between good glycemic control and renal function (41, 42), which may be altered by a toxicant induced renal impairment. The significant decrease in the plasma glucose of patients with good glycemic control compared to patients with fair or poor glycemic controls may not be unrelated to the relationship between Cd and Zn. The HbA_1c_ negatively correlates with plasma cadmium level though not significantly and suggest a trend that may be significant with larger population. Data from this study also revealed that microalbuminuria was higher in the diabetics with poor glycemic control compared to those with good glycemic control. This may also allude to a role for the environmental pollutant Cd. A better glycemic control is helpful in the prevention of nephropathy and other microvascular complications of DM (43). The Diabetes Control and Complications Trial (DCCT) showed that a target HbA_1c_ level of 7% (vs. 9%) over 9 years reduced the risk of microalbuminuria and macroalbuminuria by 34 and 56%, respectively (44). Thus, a good glycemic control may have the benefit of reducing microalbuminuria in diabetes, which may in turn be related to the stoichiometric relationship between Cd and Zn. There appears to be a deterioration of glycemic control with rise in cadmium level, as suggested by the lower cadmium level in participants with good glycemic control compared to the fair glycemic and poor glycemic control in this study.

## Conclusion

This report presents evidence of renal function deterioration in diabetes, as evidenced by the decreased urinary creatinine and increased microalbuminuria and albumin/creatinine ratio. Glycemic control may be affected with rise in cadmium level. Thus, a good glycemic control may be helpful in the prevention of nephropathy and other microvascular complications of DM. This may be achievable by reducing Cd exposure and/or by modulating the stoichiometric relationship between Cd and Zn, which appears to be a critical factor in appropriate management of MEHHs. This study therefore suggests that routine assessment of kidney function should be included in the monitoring of diabetics. Considering the low concentrations of blood Zn levels found in the diabetic patients examined in this study, a Zn supplementation may provide a significant protection against diabetes-induced complications.

### Limitation

The small sample size of the current study may in part be contributory to the equivocal findings in certain respect in this study. A larger sample size may bring out the true situation of the role of this environmental pollutant on metabolic diseases and in organs that have metabolic roles and lead to metabolic disorders when diseased, such as the kidney.

## Author Contributions

All authors listed, have made substantial, direct and intellectual contribution to the work, and approved it for publication.

## Conflict of Interest Statement

The authors declare that the research was conducted in the absence of any commercial or financial relationships that could be construed as a potential conflict of interest.
